# Measurement of complement receptor 1 on neutrophils in bacterial and viral pneumonia

**DOI:** 10.1186/1471-2334-6-11

**Published:** 2006-01-24

**Authors:** Ulla Hohenthal, Jari Nuutila, Esa-Matti Lilius, Iina Laitinen, Jukka Nikoskelainen, Pirkko Kotilainen

**Affiliations:** 1 Department of Medicine, Turku University Hospital, Kiinamyllynkatu 4–8, 20520 Turku, Finland; 2 Department of Biochemistry, University of Turku, Turku, Finland

## Abstract

**Background:**

A reliable prediction of the causative agent of community-acquired pneumonia (CAP) is not possible based on clinical features. Our aim was to test, whether the measurement of the expression of complement receptors or Fcγ receptors on neutrophils and monocytes would be a useful preliminary test to differentiate between bacterial and viral pneumonia.

**Methods:**

Sixty-eight patients with CAP were studied prospectively. Thirteen patients had pneumococcal pneumonia; 13 patients, influenza A pneumonia; 5 patients, atypical pneumonia, and 37 patients, aetiologically undefined pneumonia. Leukocyte receptor expression was measured within 2 days of hospital admission.

**Results:**

The mean expression of complement receptor 1 (CR1) on neutrophils was significantly higher in the patients with pneumococcal pneumonia than in those with influenza A pneumonia. The mean expression of CR1 was also significantly higher in aetiologically undefined pneumonia than in influenza A pneumonia, but there was no difference between pneumococcal and undefined pneumonia.

**Conclusion:**

Our results suggest that the expression of CR1 is higher in classical bacterial pneumonia than in viral pneumonia. Determination of the expression of CR1 may be of value as an additional rapid tool in the aetiological diagnosis, bacterial or viral infection, of CAP. These results are preliminary and more research is needed to assess the utility of this new method in the diagnostics of pneumonia.

## Background

Community-acquired pneumonia (CAP) is a common illness with a wide range of causative agents. The medical history of the patient and the clinical findings may be suggestive for the aetiology of CAP. In most cases, however, a reliable prediction of the causative agent of CAP is not possible on grounds of clinical features [1,2]. Neither are the manifestations on a chest radiograph specific enough for the aetiological diagnosis of CAP [2,3]. On admission, it may even be difficult to differentiate between the bacterial and viral aetiology of pneumonia. Although there is some evidence suggesting that the serum C-reactive protein (CRP) concentration is higher in pneumonias caused by *Streptococcus pneumoniae *or *Legionella pneumophila *than in those caused by other agents, the relation of CRP to the aetiology of pneumonia is controversial [4-7].

Phagocytosis is an important part of the cellular defence system to eliminate the extracellular microorganisms. The first step in the phagocytosis is adherence of a particle onto a phagocyte membrane via complement receptors and/or Fc-receptors. The aim of the present study was to examine, whether the measurement of the expression of complement receptors (CR1 and CR3) and Fcγ-receptors (FcγRI, FcγRII, and FcγRIII) on neutrophils or monocytes would be of value in differentiating between bacterial and viral pneumonia.

## Methods

Sixty-eight immunocompetent adults admitted for CAP to the Department of Medicine, Infectious Diseases Unit, Turku University Hospital, Turku, Finland, were studied prospectively. The diagnosis of pneumonia was based on the presence of an infiltrate on chest radiograph in association with fever and/or respiratory symptoms for which no other cause was found. The diagnosis of pneumonia was made by 2 of the authors (UH and PK) by consensus. In addition, the chest X rays were examined by a specialist in radiology.

The mean age of the patients was 53.7 years (range, 18 to 87 years). There were 41 males and 27 females. Underlying diseases were present in 28 patients; COPD, cardiovascular disease and alcoholism being the most common. None of the patients needed mechanical ventilation or treatment in the intensive care unit. All patients gave a written consent, which was approved by the institutional ethics committee.

For each patient, the microbiological examinations and the treatment were carried out according to the routine clinical practice [8,9]. *Mycoplasma pneumoniae *and *Chlamydia pneumoniae *IgG and IgM antibodies were measured by commercial enzyme immunoassay kits (IgG-EIA, and IgM-EIA, ThermoLabsystems, Helsinki, Finland) [10] according to the instructions of the manufacturer; and *Legionella *IgG and IgM antibodies, using a previously described method, with *L. pneumophila *1–4 and *L. micdadei *as antigens [11]. Serology for respiratory viruses (influenza A and B viruses, adenovirus, respiratory syncytial, parainfluenza virus types 1, 2 and 3) was done with antigens prepared at the Department of Virology, University of Turku, by enzyme immunoassay as described previously [12]. Viral antigens for respiratory viruses were detected by time-resolved fluoroimmunoassay [13]. *M. pneumoniae *polymerase chain reaction (PCR) test and *Legionella *spp. PCR test were performed as described previously [14,15]. Unconcentrated urine samples were tested using the immunochromatographic assay Binax NOW *S. pneumoniae *antigen (Binax, Portland, Maine).

Isolation of *S. pneumoniae *from blood cultures or detection of pneumococcal capsular antigen in urine was considered diagnostic for *S. pneumoniae*. A 4-fold or greater increase in serologic titres was considered diagnostic for *M. pneumoniae*, *C. pneumoniae*, or *L. pneumophila*. Identification of specific DNA for *M. pneumoniae *or *L. pneumophila *in a throat swab or sputum sample made a definitive causative diagnosis. Recognition of respiratory viruses was based on the detection of viral antigen in the nasopharyngeal sample or a 4-fold or greater increase in serologic titres.

For the measurement of leukocyte receptor expression, 10 ml of heparin anticoagulated blood was collected from the patients within 2 days of hospital admission. The procedure was performed as described previously using fluorescence-labelled receptor-specific monoclonal antibodies [16]. FITC-conjugated anti-FcγRI (CD64; mouse IgG1 isotype, clone 22), anti-FcγRIII (CD16; mouse IgG1 isotype, clone 3G8), anti-CR1 (CD35; mouse IgG1 isotype, clone J3D3), and mouse IgG1 isotype control (clone 679.1Mc7) as well as PE-conjugated anti-FcγRII (CD32; mouse IgG2a isotype, clone 2E1), anti-CR3 (CD11b; mouse IgG1 isotype, clone Bear1), mouse IgG1 isotype control (clone 679.1Mc7), and mouse IgG2a isotype control (clone U7.27) were purchased from Immunotech (Marseille, France). A relative measure of receptor expression was obtained by determining the mean fluorescence intensity (MFI) of 5000 leukocytes by flow cytometer.

Concurrently with the collection of blood for the measurement of leukocyte receptor expression, blood or plasma samples were taken for the measurement of CRP, erythrocyte sedimentation rate (ESR), and white blood cell count (WBC). The expression of leukocyte receptors in pneumonia patients was compared to the expression of leukocyte receptors in 63 healthy controls. CRP and ESR values were not analysed for the controls.

All data in Table 1 and box chart presentation (Figure) are expressed as the mean (SD). In the box chart, 25%, 50%, and 75% quartiles are also presented. First, the group differences were tested using analysis of variance (ANOVA). Pairwise, group comparisons after ANOVA were carried out using Tukey's multiple comparison technique. A p-value of less than 0.05 was considered significant.

**Table 1:** Comparison of receptor expressions of neutrophils and monocytes in patients with *Streptococcus pneumoniae*, influenza A and aetiologically undefined pneumonia. P values for overall group differences tested using analysis of variance (ANOVA).

Parameter	A *S. pneumoniae * (n = 13)	B Influenza A (n = 13)	C Undefined (n = 37)	D Control (n = 63)	p value
	Mean (SD)	Mean (SD)	Mean (SD)	Mean (SD)	
WBC (×10^9^/l)	12.3 (6.9)	7.6 (2.2)	10.3 (4.2)	4.8 (1.3)	<0.0001
PMNL (×10^9^/l)	8.9 (4.4)	5.5 (2.3)	7.5 (3.6)	2.6 (0.9)	<0.0001
CRP (mg/l)	370.0 (121.5)	108.2 (87.7)	179.7 (94.4)	-	<0.0001
ESR (mm/h)	79.4 (20.4)	39.1 (29.7)	69.2 (24.1)	-	0.0018
					
**Receptor expression of neutrophils**					
CR1	20.4 (9.8)	7.5 (4.6)	19.3 (8.8)	6.3 (2.2)	<0.0001
CR3	63.1 (32.6)	80.7 (40.9)	107.2 (47.9)	48.5 (17.8)	<0.0001
FcγRI	4.7 (2.5)	2.9 (2.5)	5.0 (5.3)	0.6 (0.3)	<0.0001
FcγRII	12.8 (5.4)	10.3 (4.2)	13.0 (4.3)	11.0 (1.8)	0.0350
FCγRIII	89.6 (43.3)	111.7 (46.2)	120.3 (43.1)	128.0 (34.3)	0.0285
					
**Receptor expression of monocytes**					
CR1	11.7 (4.5)	9.7 (5.0)	15.3 (6.7)	4.6 (2.7)	<0.0001
CR3	63.9 (30.8)	84.6 (30.4)	109.8 (65.1)	44.6 (33.5)	<0.0001
FcγRI	14.0 (3.7)	18.9 (4.5)	18.9 (8.7)	8.4 (1.9)	<0.0001
FcγRII	13.1 (6.4)	18.5 (6.9)	18.7 (8.2)	11.7 (1.9)	<0.0001

WBC = white blood cell count, PMNL = polymorphonuclear leukocyte count, CRP = serum C-reactive protein, ESR = erythrocyte sedimentation rate.

## Results

An aetiological agent was established in 30 of the 68 patients with CAP. Group A consisted of 13 patients with *S. pneumoniae *infection (10 detected by blood culture, 3 by pneumococcal antigen test) and group B consisted of 13 patients with influenza A infection (11 identified by viral antigen detection on the nasopharyngeal sample, and 2 by serology). In group C, the aetiological agent of the 37 patients remained unknown. Atypical pneumonia was identified in 5 patients: 3 *M. pneumoniae *by serology, 1 *C. pneumoniae *by serology, and 1 *L. pneumophila *by PCR and serology.

CRP and ESR were significantly higher in the patients with pneumococcal or aetiologically undefined pneumonia than in those with influenza A pneumonia (Tables 1 and 2). CRP was also significantly higher in pneumococcal pneumonia than in aetiologically undefined pneumonia. Total WBC was significantly lower in influenza A pneumonia than in aetiologically undefined pneumonia, but there was no difference in WBC between influenza A pneumonia and pneumococcal pneumonia.

**Table 2:** Comparison of receptor expressions of neutrophils and monocytes in patients with *Streptococcus pneumoniae*, influenza A and aetiologically undefined pneumonia. Group comparisons after ANOVA carried out using Tukey's multiple comparison technique.

	p value					
	
Parameter	A vs B	A vs C	B vs C	A vs D	B vs D	C vs D
WBC (×10^9^/l)	0.1052	0.7702	0.0243	0.0010	<0.0001	<0.0001
PMNL (×10^9^/l)	0.0586	0.7265	0.0813	<0.0001	0.0001	<0.0001
CRP (mg/l)	<0.0001	<0.0001	0.0417	-	-	-
ESR (mm/h)	0.0012	0.3333	0.0094	-	-	-
						
**Receptor expression of neutrophils**						
CR1	0.0002	0.9836	<0.0001	<0.0001	0.8091	<0.0001
CR3	0.6191	0.0020	0.2263	0.3983	0.0307	<0.0001
FcγRI	0.2826	0.9935	0.2518	<0.0001	0.0053	<0.0001
FcγRII	0.5413	0.9998	0.2031	0.6273	0.9311	0.0431
FCγRIII	0.6049	0.1487	0.9358	0.0222	0.6231	0.7949
						
**Receptor expression of monocytes**						
CR1	0.7146	0.1320	0.0100	<0.0001	0.0027	<0.0001
CR3	0.3126	0.0057	0.2553	0.1858	0.0002	<0.0001
FcγRI	0.0166	0.0308	1.0000	<0.0001	<0.0001	<0.0001
FcγRII	0.1630	0.0655	0.9999	0.8781	0.0033	<0.0001

WBC = white blood cell count, PMNL = polymorphonuclear leukocyte count, CRP = serum C-reactive protein, ESR = erythrocyte sedimentation rate, A = *S. pneumoniae *pneumonia, B = Influenza A pneumonia, C = Aetiologically undefined pneumonia, D = controls.

The mean expression of CR1 on neutrophils was significantly higher in the patients with pneumococcal pneumonia than in those with influenza A pneumonia. The mean expression of CR1 was also significantly higher in aetiologically undefined pneumonia than in influenza A pneumonia, but there was no difference between pneumococcal and undefined pneumonia. Patients with influenza A could be divided in 2 subgroups by the expression of CR1 on neutrophils: 8 patients with CR1 ranging from 2.09 to 5.32 and 5 patients with CR1 ranging from 11.1 to 15.5. The CRP values ranged from 44 to 178 mg/l and from 120 to 300 mg/l in these subgroups, respectively. The expression of neutrophil CR1 in the patients and controls is presented in the Figure.

The number of the patients with atypical pneumonia was too small to be included in the statistical analysis. The expression of CR1 ranged from 3.3 to 8.6 in the 3 patients with *M. pneumoniae *infection, was 6.8 in the patient with *C. pneumoniae *infection, and 33.6 in the patient with *L. pneumophila *infection.

A significant difference in the expression of monocyte FcγRI was observed between the patients with pneumococcal and influenza A pneumonia, and in the expression of monocyte CR1 between the patients with influenza A and undefined pneumonia.

The results of the receptor expression of neutrophils and monocytes of the patients and controls are presented in Table 1.

## Discussion

The main finding of the present study was that the expression of CR1 on neutrophils was significantly higher in the patients with pneumococcal pneumonia than in those with influenza A pneumonia. On a more general level, this suggests that the expression of neutrophil CR1 is higher in classical bacterial pneumonia than in viral pneumonia. The high level of CR1 in aetiologically undefined pneumonia is consistent with this finding, since one can speculate on epidemiological basis [1,17] that most of these patients probably had bacterial pneumonia. In the patients with *M. pneumoniae *and *C. pneumoniae *infection, the expression of CR1 was low, though their number was too small to allow any distinct conclusions to be drawn from this finding. The subgroup of patients with influenza A pneumonia, who had CR1 levels ≥ 11.1, is of note, but could be explained by e.g. concomitant bacterial pneumonia.

Also other significant differences were observed. The expression of monocyte FcγRI was significantly higher in the patients with influenza A pneumonia than in those with pneumococcal pneumonia, but no difference was found between influenza A and aetiologically unidentified pneumonia. On the other hand, the expression of monocyte CR1 was significantly higher in the patients with unidentified pneumonia than in those with influenza A pneumonia, but no difference was found between pneumococcal and influenza A pneumonia. Thus, these results imply that the expression of neutrophil CR1 may be associated with a better ability than that of the other the neutrophil receptors, or of the monocyte receptors, to differentiate between the bacterial and viral aetiology of pneumonia.

The behaviour of CRP was similar to the expression of neutrophil CR1 in that CRP was significantly higher in pneumococcal and aetiologically undefined pneumonia than in influenza A pneumonia. In a previous study by García Vázquez et al. [5], however, the mean CRP values were not significantly different among pneumonias caused by agents other than *L. pneumophila*.

Although the high expression of neutrophil CR1 is suggestive of classical bacterial pneumonia, it is unlikely that any single parameter of inflammation alone could reliably differentiate between bacterial and viral pneumonia. Rather, it is possible that the diagnostic accuracy could be improved by combination of the results of CRP, ESR, and several cell receptors. Studies are presently underway to determine, whether the diagnostic yield provided by the measured individual variables would increase upon combination.

## Conclusion

Our results suggest that the expression of neutrophil CR1 is higher in classical bacterial pneumonia than in viral pneumonia. Based on these findings, we suggest that determination of the expression of CR1 on neutrophils may be of value as an additional rapid tool in the aetiological diagnosis, bacterial or viral infection, of CAP. Our results are preliminary and more research is needed to assess the utility of this new method in the diagnostics of pneumonia.

## Competing interests

The author(s) declare that they have no competing interests.

## Authors' contributions

All authors planned and carried out the conception and design of the study. UH and PK were involved in patient care, and acquisition of data. JNu, IL, and EL were responsible for the measurement of leukocyte receptor expression in the laboratory, and all authors were responsible for interpretation of the data. UH wrote the first draft of the manuscript, and all authors participated in its revision. All authors had intellectual contribution, and all read and approved the final manuscript.

**Figure 1:**
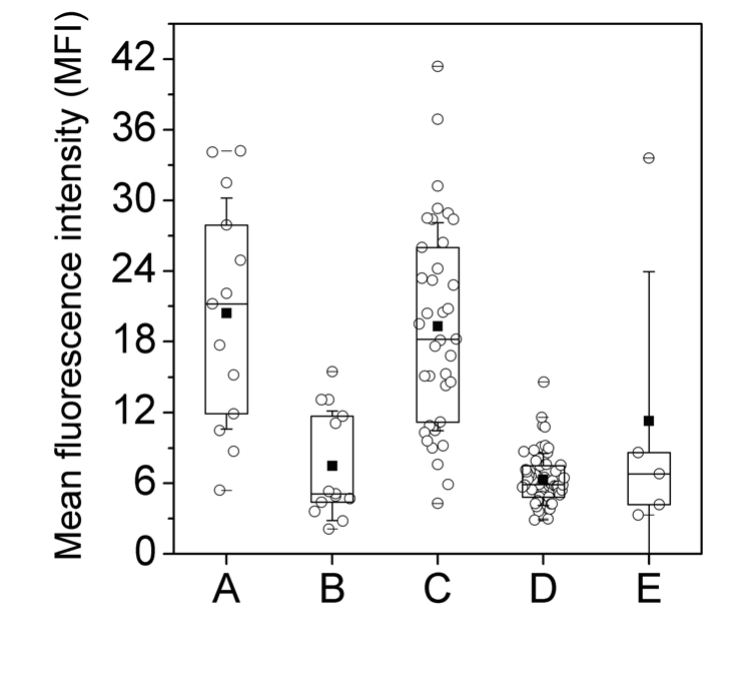
**The expression of CR1 on neutrophils in patients with pneumonia and controls**. A: *Streptococcus pneumoniae *pneumonia; B: influenza A pneumonia; C: aetiologically undefined pneumonia; D: controls; E: atypical pneumonia.

## Pre-publication history

The pre-publication history for this paper can be accessed here:


